# New-onset atrial fibrillation following arteriovenous fistula increases adverse clinical events in dialysis patients with end-stage renal disease

**DOI:** 10.3389/fcvm.2024.1386304

**Published:** 2024-04-12

**Authors:** Wenhui Song, Lizhou Wu, Chong Sun, Xianglei Kong, Haiyan Wang

**Affiliations:** ^1^ Department of Medical Ultrasound, Shandong Medicine and Health Key Laboratory of Abdominal Medical Imaging, The First Affiliated Hospital of Shandong First Medical University & Shandong Provincial Qianfoshan Hospital, Jinan, China; ^2^ Department of Nephrology, Shandong Key Laboratory of Rheumatic Disease and Translational Medicine, The First Affiliated Hospital of Shandong First Medical University & Shandong Provincial Qianfoshan Hospital, Shandong Institute of Nephrology, Jinan, China

**Keywords:** maintenance haemodialysis, autologous arteriovenous fistula, AF, echocardiography, end-stage renal disease (ESRD)

## Abstract

**Background:**

End-stage renal disease (ESRD) patients have a high potential cardiovascular burden, and cardiovascular disease (CVD) is the leading cause of death in maintenance haemodialysis (MHD) patients. Arteriovenous fistula (AVF) is the preferred vascular access for MHD patients, but AVF significantly affects the haemodynamics of the cardiovascular system, leading to or exacerbating CVD, including atrial fibrillation (AF). This study aimed to evaluate the impact of AVF on cardiac function, especially of the left atrium (LA), in patients with ESRD and to further explore the relationship between AVF establishment and the occurrence of AF.

**Methods:**

We selected 1,107 ESRD patients on haemodialysis using AVF and 550 patients with tunneled-cuffed catheters (TCC) admitted between January 2016 and December 2022 for follow-up to compare the rate of AF between the two groups. A total of 153 patients in the AVF group with complete information (clinical data, echocardiographic and biochemical indices, and other data) were enrolled and retrospectively analysed for risk factors for the development of AF and were followed up for adverse clinical outcomes (including all-cause death, cardiac death, readmission due to heart failure, and stroke).

**Results:**

The incidence of new-onset AF was higher in the AVF group than the TCC group after dialysis access was established (16.30% vs. 5.08%, *P* < 0.001). Echocardiography showed that the LA anteroposterior diameter increased (*P* < 0.001) and the incidence of AF increased from 11.76% to 26.14% (*P* = 0.001) after AVF establishment. Multivariate logistic regression analysis showed that age and LA enlargement were independent risk factors for new-onset AF after AVF establishment (*P* < 0.05). Adverse clinical outcomes were more common in patients with AF than in patients without AF (*P* < 0.001). Multivariate Cox risk regression analysis suggested that new-onset AF (HR = 4.08, 95% CI: 2.00–8.34, *P* < 0.001) and left ventricular systolic dysfunction (HR = 2.42, 95% CI: 1.20–4.88, *P* = 0.01) after AVF establishment were independent risk factors for adverse clinical outcomes.

**Conclusion:**

LA enlargement after AVF establishment is associated with a significant increase in the incidence of AF, in addition, AF which is as an important influential factor in patients with MHD combined other systemic diseases might increase adverse clinical events.

**Clinical Trial Registration:**

(NCT 06199609)

## Background

In recent years, the total number of patients receiving treatment for end-stage renal disease (ESRD) worldwide has been steadily increasing, with a growth rate exceeding the population growth rate (1). Common renal replacement therapies include dialysis and kidney transplantation, but due to donor limitations, maintenance haemodialysis (MHD) remains the preferred treatment for most ESRD patients. ESRD patients have a high potential cardiovascular burden, and cardiovascular disease (CVD) is the leading cause of death in MHD patients (2). Arteriovenous fistula (AVF) is the preferred vascular access for MHD patients due to its safety, convenience, low complication rate, and high long-term patency (1). However, AVF significantly affects the haemodynamics of the cardiovascular system, leading to or exacerbating the occurrence of CVD, including left ventricular (LV) hypertrophy, heart failure, pulmonary hypertension (PH), and valve dysfunction (3). There have been relatively few long-term follow-up cohort studies on cardiac structure and function after AVF establishment, and most have focused on the association between AVF and LV dysfunction. Clinically, the incidence of atrial fibrillation (AF) in MHD patients undergoing AVF dialysis also appears to be relatively high. AF increases the risk of adverse clinical outcomes such as stroke, systemic arterial embolism, dementia, heart failure, myocardial infarction, venous thromboembolism, and sudden death (4), but there have been no published studies on the relationship between AVF establishment and AF.

This retrospective cohort study aimed to evaluate the impact of AVF on the heart, especially the LA, of MHD patients and to further explore the relationship between AVF establishment and the occurrence of AF and adverse clinical outcomes, providing a theoretical basis for cardioprotective measures.

## Materials and methods

### Study population

This was a single-centre retrospective cohort study in which patients who received long-term MHD from January 2016 to December 2022 were retrospectively enrolled. The inclusion criteria were patients (1) with end-stage renal disease receiving haemodialysis, with an eGFR <15 ml/min/1.73m2; (2) aged > 18 years; (3) receiving regular dialysis; (4) with AVF or TCC as the sole vascular access; and (5) with AVF flow rates between 0.5-1.0 L/min. The exclusion criteria were as follows: (1) AVF flow > 1.0 L/min; (2) combined heart disease such as severe valvular disease, intracardiac shunts, constrictive pericarditis, or postcardiac transplantation; (3) previous allogeneic kidney transplantation or peritoneal dialysis; (4) discontinued dialysis due to AVF failure; (5) risk factor analysis for new-onset AF, AF and LA enlargement before AVF establishment; and (6) survival analysis and Cox regression analysis of adverse clinical outcomes between the AF group and the non-AF group. Patients with AF before AVF establishment were excluded.

### Methods

#### Differences in haemodialysis access

Patients in the AVF group underwent AVF creation by anastomosing the radial artery in the anterior forearm with the cephalic vein. Patients in the TCC group had a tunnelled cuffed catheter inserted via the internal jugular vein for haemodialysis.

#### Final study population

Patients in the AVF group with complete medical records within 3 months before and after AVF establishment were enrolled to facilitate long-term, longitudinal evaluation of cardiac structure and function. Clinical data and laboratory and echocardiographic findings were collected from the hospital's electronic medical records system.

#### AF detection approach

In this study, AF was mainly detected through surface electrocardiograms, dynamic electrocardiograms, and smart wearable devices at home.

#### Echocardiography examination and measurement

Echocardiography was conducted by an ultrasound diagnostic instrument (PHILIPS Epiq 7c, USA) with an S5-1 cardiac probe operating at a frequency of 2–4 MHz (5). The following parameters were measured: LA anteroposterior diameter (LAD), LA volume (LAV), right atrial diameter (RAD), interventricular septum thickness (IVST), LV posterior wall thickness (LVPW), LV end-diastolic diameter (LVDD), right ventricular end-diastolic diameter (RVDD), and LV ejection fraction (LVEF). The LA volume index was determined using the biplane disc method, and the LV mass index (LVMI) was calculated as LV mass (LVM) divided by body surface area (BSA) (LVMI = LVM/BSA). We assessed pulmonary arterial hypertension followed the guidelines of the American Society of Echocardiography (6), defining PH as pulmonary arterial systolic pressure (PASP) ≥ 35 mmHg (1 mmHg = 0.133 kPa). LA enlargement was defined as LAD >38 mm (males) and LAD >37 mm (females). LV systolic dysfunction was defined as an LVEF <52% (males) or <53% (females) (5).

#### Primary endpoint and secondary endpoint

The primary endpoint events were defined as all-cause mortality and cardiovascular mortality, while the secondary endpoint events included heart failure readmission and stroke. Patients who underwent kidney transplantation, who changed dialysis methods during follow-up, or who did not experience endpoint events at the end of follow-up were censored.

### Statistical analysis

Data analysis was performed using SPSS 26.0. For normally distributed continuous data, the results are expressed as mean ± standard deviation (SD), while skewed distributed data are expressed as M (P25, P75). Group differences were examined using the chi-square test, *t*-test, or Wilcoxon rank-sum test. The paired-sample t test, Wilcoxon rank-sum tests, or paired chi-square test was used to compare data before and after AVF establishment. Binary logistic regression analysis was employed to assess the risk factors for new-onset AF after AVF establishment. Kaplan-Meier survival curve analysis was used to compare differences in clinical outcomes between groups. Single-factor Cox regression analysis was used to select variables with *P* < 0.10 for multifactorial Cox regression analysis to evaluate the risk factors for adverse clinical outcomes. The results are expressed as hazard ratios (HRs) and their 95% confidence intervals (95% CIs) and were analysed using a two-tailed test, with *P* < 0.05 considered statistically significant.

## Results

This study included a total of 1,657 ESRD patients, with 1,107 in the AVF group and 550 in the TCC group. The complete medical data of 153 patients in the AVF group were further analysed in detail. Complete data were defined complete echocardiography data, laboratory indicators, and general clinical data both before and after AVF establishment. Among the 1,657 ESRD patients initially included, 1,107 patients underwent AVF haemodialysis, but most patients underwent only AVF surgery at our hospital, which is technically difficult. Their long-term haemodialysis was performed in local hospitals, and medical information for these patients was difficult to collect. Among them, only 153 AVF patients who agreed to long-term dialysis at our hospital with complete medical data were included in this study.

### Comparison of AF incidence between the AVF group and TCC group

The incidence of AF in the TCC group was higher than that in the AVF group before the establishment of haemodialysis access (14.18% vs. 10.30%, *P* = 0.020). Follow-up after haemodialysis revealed a higher incidence of new-onset AF in the AVF group than in the TCC group (16.30% vs. 5.08%, *P* < 0.001). The median follow-up time was 18 (3, 60) months for the AVF group and 22 (4, 58) months for the TCC group.

### Baseline clinical characteristics

The 153 patients with complete medical data in the AVF group were compared with the excluded subjects who had AVF but underwent haemodialysis elsewhere (*n* = 954). The baseline characteristics were similar (Table 1). The 153 patients were characterized by advanced age, overweight, anaemia, and multiple comorbidities, such as diabetes, hypertension, and heart failure, consistent with a typical ESRD population (Table 2). The aetiology of ESRD was approximately 50.00% diabetic nephropathy, 25.00% for chronic glomerulonephritis, and 25.00% for other causes (hypertension, polycystic kidney disease, and immune-related factors). After AVF establishment, BMI and blood pressure decreased, and heart rate and incidence of AF increased.

**Table 1:** Comparison of baseline information of the study population and the remaining samples excluding the study subjects.

	Study population (*n* = 153)	The excluded subjects (n = 954)	*P*-value
Demographics			
Age of initiation of dialysis, years	59 (50, 66)	61 (53, 67)	0.19
Male, *n* (%)	116 (75.82%)	677 (70.96%)	0.23
BMI, Kg/m^2^	25.71 ± 4.49	28.21 ± 4.33	0.13
Haemoglobin, g/L	87 (73, 99)	89 (78, 102)	0.21
Calcium, mg/L	2.07 (1.99, 2.16)	2.10 (1.96, 2.26)	0.16
Phosphorus, mg/L	1.66 (1.37, 1.95)	1.77 (1.42, 2.24)	0.21
Comorbidities			
Hypertension, *n* (%)	140 (91.50%)	830 (87.00%)	0.12
Diabetes, *n* (%)	85 (55.56%)	553 (57.97%)	0.58
Coronary artery disease, *n* (%)	50 (32.68%)	334 (35.01%)	0.57
Atrial fibrillation, *n* (%)	18 (11.76%)	132 (13.84%)	0.49
Echocardiograpy			
LVEF, %	61.21 ± 7.15	60.65 ± 7.34	0.50
LA enlargement, *n* (%)	35 (22.88%)	239 (25.05%)	0.56

Statistics are mean ± standard deviation, median (interquartile range) or number (%).

BMI, body mass index; LVEF, left ventricular ejection fraction.

**Table 2:** General information characteristics of the research population.

AVF creation cohort (*n* = 153)	
Demographics	
Age of initiation of dialysis, years	59 (50, 66)
Male, *n* (%)	116 (75.82%)
Female, *n* (%)	37 (24.18%)
BMI, Kg/m^2^	25.71 ± 4.49
Heart rate, bpm	80 (72, 87)
Systolic BP, mmHg	156 ± 22
Diastolic BP, mmHg	88 ± 15
Smoking, *n* (%)	53 (34.64%)
Alcohol consumption, *n* (%)	48 (31.37%)
Primary disease	
Diabetes, *n* (%)	75 (49.02%)
Chronic glomerulonephritis, *n* (%)	41 (26.80%)
Hypertension, *n* (%)	12 (7.84%)
Polycystic kidney, *n* (%)	7 (4.58%)
Other, *n* (%)	18 (11.76%)
Comorbidities	
Diabetes, *n* (%)	85 (55.56%)
Hypertension, *n* (%)	140 (91.50%)
Coronary artery disease, *n* (%)	50 (32.68%)
Heart failure, *n* (%)	32 (20.92%)
Cerebral infarction, *n* (%)	36 (23.53%)
Cerebral hemorrhage, *n* (%)	6 (3.92%)
Atrial fibrillation, *n* (%)	18 (11.76%)
Pulmonary arterial hypertension, *n* (%)	37 (24.18%)
Access site	
Left, *n* (%)	139 (90.85%)
Right, *n* (%)	14 (9.15%)
Laboratories	
Red blood cell count, 10^12/L	2.92 ± 0.63
Haemoglobin, g/L	87 (73, 99)
Albumin, g/L	34.32 ± 5.17
Urea, mmol/L	23.6 (18.2, 30.08)
Phosphorus, mg/L	1.66 (1.37, 1.95)
Calcium, mmol/L	2.07 (1.99, 2.16)
Potassium, mmol/L	5.35 (3.56, 5.66)
PTH, pg/ml	189.6 (106.7, 324.3)

Statistics are mean ± standard deviation, median [interquartile range] or number (%).

AVF, arteriovenous fistula; BMI, body mass index; PTH, parathormone.

### Management of AF in our study

In this study, no AF patients underwent electroconversion or radiofrequency ablation surgery. Approximately 80% of AF patients received anticoagulant therapy (60% of AF patients received anticoagulant combined with ventricular rate control, and approximately 40% of AF patients received anticoagulant combined with antiarrhythmic therapy).

### Echocardiographic and laboratory parameters

The median time for echocardiography re-examination after AVF establishment was 21 (7, 46.5) months. Echocardiography revealed an enlarged the LAD and RAD. There were no significant changes in LVDD or RVDD, but the LVMI increased, and the LVEF decreased. Laboratory results showed an increase in red blood cells, haemoglobin, and haematocrit (Table 3).

**Table 3:** Changes in clinical features, echocardiographic and laboratory indices before and after AVF establishment.

	Pre AVF (*n* = 153)	Post AVF (*n* = 153)	Mean difference [95% CI]	*P*-value
Vital Signs				
Heart rate, bpm	80	84	–	0.001
Systolic BP, mmHg	156	149	−7.00 (−12.91 to −2.60)	0.003
Diastolic BP, mmHg	88	84	−4.00 (−7.03 to −1.51)	0.041
BMI, Kg/m^2^	25.71	24.41	−1.30 (−2.10 to −0.51)	0.004
Atrial fibrillation, %	11.76	26.14	–	0.001
Pulmonary hypertension (%)	24.18	27.45	–	0.514
Echocardiographic data				
LA (mm)	38.11	41.70	–	<0.001
LVEDD (mm)	50.48	51.01	+0.53 (−0.46 to 1.52)	0.292
LVMI (g/m^2^)	110.97	124.56	+13.59 (1.44–25.74)	0.031
Longitudinal diameter of RA (mm)	45.50	47.34	+1.84 (0.75–2.93)	0.001
Transverse diameter of RA (mm)	37.62	39.84	–	<0.001
RVDD (mm)	23.04	23.56	–	0.105
SPAP (mmHg)	34.88	36.20	–	0.601
LVEF (%)	61.21	57.67	–	0.002
Laboratories				
Erythrocyte count, 10^12/L	2.92	3.20	+0.28 (0.12–0.49)	0.002
Hemoglobin, g/L	87	99.50	–	<0.001
Hematocrit	0.27	0.30	+0.03 (0.01–0.05)	0.001

Values represent paired *t*-test, Wilcoxon rank sum test or paired chi square test. *P*-values are not adjusted for multiple comparisons.

CI, confidence interval; BMI, body mass index; LA, left atrial; LV, left ventricle; LVEDD, left ventricular end diastolic dimension; RA, right atrium; RV, right ventricle; RVDD, right ventricular end diastolic diameter; LVEF, left ventricular ejection fraction.

### Age and LA enlargement as independent risk factors for new-onset AF after AVF establishment

To clarify the relationship between LA enlargement after AVF establishment and the onset of new AF, 102 patients with pre-existing AF (*n* = 18) and those with LA enlargement (*n* = 33) before AVF establishment were excluded. These 102 patients were divided into the LA enlargement group (*n* = 62) and the normal LA group (*n* = 40), and the incidence of new-onset AF was 20.97% and 5.00%, respectively (*P* = 0.026).

**Figure 1:**
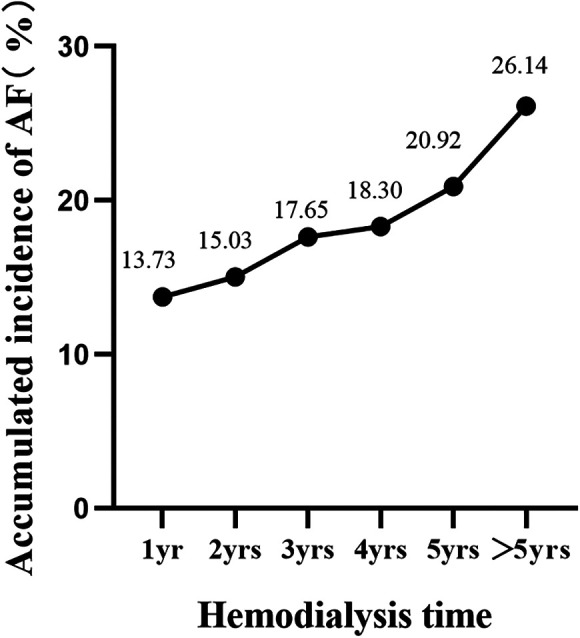
Accumulated incidence of AF with hemodialysis time.

Univariate binary logistic analysis revealed no statistically significant differences in sex, concomitant hypertension, diabetes, or hyperlipidaemia (*P* > 0.05) but did reveal statistically significant differences in age, serum potassium, and LA enlargement (*P* < 0.05) between patients with and without new-onset AF after AVF establishment. Multivariate logistic regression analysis showed that age (OR 1.09, 95% CI 1.03–1.17) and LA enlargement (OR 5.12, 95% CI 1.04–25.79) were independent risk factors for new-onset AF after AVF establishment (*P* < 0.05) (Table 4). The dialysis time in this study was 59 (34, 82) months, and both univariate and multivariate logistic regression showed that the duration of dialysis was not an independent risk factor for newly developed AF. However, with longer dialysis time, the cumulative incidence of AF rose (Figure 1).

**Table 4:** Logistic regression analysis affecting new-onset AF in patients after AVF establishment.

	Univariate			Multivariate		
	*P*	OR	95%CI	*P*	OR	95%CI
Genders	0.49	0.71	0.26–1.91			
Age of initiation of dialysis, years	<0.001	1.09	1.04–1.15	0.01	1.09	1.03–1.17
Hypertension	0.22	0.40	0.10–1.71			
Diabetes	0.13	2.20	0.80–6.10			
High blood fat disease	0.90	0.88	0.10–7.70			
Hemoglobin	0.18	0.98	0.96–1.00			
Calcium	0.75	0.67	0.06–8.11			
Phosphorus	0.52	1.26	0.23–1.53			
Potassium	0.02	3.24	1.22–8.57	0.70	1.29	0.36–4.67
PTH	0.08	1.00	0.99–1.01			
Hemodialysis time	0.058	0.98	0.96–1.00	0.07	0.98	0.95–1.00
Left atrial enlargement after AVF establishment	0.030	5.53	1.18–25.99	0.03	5.86	1.14–29.98

AVF, arteriovenous fistula; PTH, parathormone.

### Increased incidence of adverse clinical outcomes in the AF group

**Figure 2:**
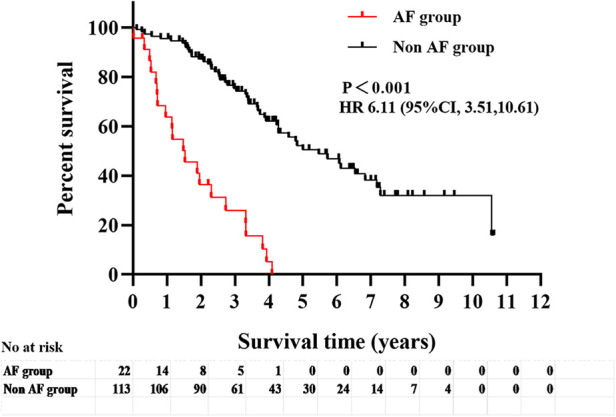
Analysis of survival curves of adverse clinical outcomes (all-cause mortality, cardiovascular mortality, heart failure readmission and stroke) in the two groups with or without comorbid AF.

After excluding patients with a history of AF before AVF establishment (*n* = 18), the other patients (*n* = 135) were divided into the AF group (*n* = 22) and the non-AF group (*n* = 113), and their clinical outcomes were compared during follow-up. The follow-up period of this study was 40 (25.5, 65.5) months. The AF group met a total of 20 endpoint events (90.90%), including 16 cases of all-cause mortality (80.00%) (of which 37.50% were due to cardiac causes), 2 cases of stroke (10.00%), and 2 cases of rehospitalization due to heart failure (10.00%). The non-AF group had a total of 52 endpoint events (46.00%), including 33 cases of all-cause mortality (63.46%) (of which 42.42% were due to cardiac causes), 4 cases of stroke (7.69%), and 15 cases of rehospitalization due to heart failure (28.85%). Kaplan-Meier survival analysis of the two groups showed a significant difference in the survival curves (*P* < 0.001), indicating a higher incidence of adverse clinical outcomes in the AF group than in the non-AF group (Figure 2).

### New-onset AF and LV systolic dysfunction as risk factors for adverse clinical outcomes

By the end of the follow-up, after excluding patients who had AF before AVF establishment, among the remaining 135 patients, 49 patients (36.3%) experienced major endpoint events, with 29 patients (59.18%) experiencing all-cause mortality and 20 patients (40.82%) experiencing cardiovascular mortality. Additionally, 23 patients (17.0%) experienced minor endpoint events. Single-factor Cox regression analysis indicated that age, diastolic blood pressure, comorbid diabetes, comorbid coronary heart disease, preoperative haemoglobin level, new-onset AF, and LV systolic dysfunction after AVF establishment were risk factors for adverse clinical outcomes (*P* < 0.10). After multivariate Cox regression analysis, it was suggested that new-onset AF (HR = 4.08, 95% CI: 2.00–8.34, *P* < 0.001) and LV systolic dysfunction (HR = 2.42, 95% CI: 1.20–4.88, *P* = 0.01) were independent risk factors for adverse clinical outcomes in AVF patients (Table 5).

**Table 5:** Univariate and multivariate COX proportional risk regression model analysis of the occurrence of adverse clinical outcome events in AVF patients (*n* = 135).

	Univariate			Multivariate		
	*P*	HR	95%CI	*P*	HR	95%CI
Genders	0.57	0.86	0.53–1.44			
Age of initiation of dialysis, years	<0.001	1.04	1.02–1.06	0.15	1.01	0.99–1.04
BMI, Kg/m^2^	0.29	1.11	0.91–1.35			
Hypertension	0.07	0.49	0.22–1.07			
Diabetes	0.001	2.50	1.49–4.21	0.65	1.16	0.61–2.21
Coronary artery disease	<0.001	3.21	1.98–5.20	0.09	1.73	0.93–3.33
Smoking	0.88	1.04	0.64–1.69			
Drinking	0.40	1.25	0.75–2.09			
LVMI (g/m^2^)	0.52	1.03	0.95–1.11			
LVEF, %	0.50	0.99	0.96–1.02			
Hemoglobin, g/L	0.03	0.56	0.33–0.96	0.52	0.81	0.43–1.53
Calcium, mmol/L	0.05	5.03	0.98–25.79			
Phosphorus, mmol/L	0.54	0.88	0.58–1.33			
Changes after AVF establishment						
New-onset atrial fibrillation	<0.001	6.11	3.51–10.61	<0.001	4.08	2.00–8.34
left atrial enlargement	0.49	0.85	0.53–1.34			
Left ventricular systolic dysfunction	0.001	2.59	1.49–4.47	0.01	2.42	1.20–4.88

BMI, Body Mass Index; LAVI, left atrial volume index; LV, left ventricle; LVEF, left ventricular ejection fraction.

## Discussion

Haemodialysis is the preferred treatment for ESRD patients, over 70% them undergoing MHD treatment. Previous studies have reported a wide variation in the prevalence of AF among MHD patients, ranging from 2.8% to 27% (7–10), far higher than that among the general population. This study found that (1) haemodialysis patients with AVF have a significantly higher incidence of new-onset AF in than those with TCC. (2) Echocardiography revealed LA enlargement, increased LVMI, decreased LVEF, and a further increase in the occurrence of AF after AVF establishment. (3) Multivariate logistic regression analysis indicated that age and LA enlargement are independent risk factors for new-onset AF. (4) There were more adverse clinical outcomes in AF patients than in non-AF patients. (5) Multifactorial Cox regression analysis suggested that new-onset AF after AVF establishment and LV systolic dysfunction are independent risk factors for adverse clinical outcomes in MHD patients.

An AVF is a nonphysiological shunt established between the high-pressure, high-resistance arterial system and the low-pressure, high-capacity venous system. On the one hand, it increases the volume of blood returning to the heart, thereby increasing the cardiac volume load. According to Frank-Starling's law, a compensatory increase in myocardial contractility and stroke volume leads to myocardial hypertrophy, ischaemia, and fibrosis and subsequently induces cardiac dysfunction. Higher volume load will also lead to elevated ventricular filling pressure and diastolic dysfunction. On the other hand, long-term hypertension and endothelial dysfunction increase peripheral resistance, decrease arterial compliance, and aggravate the pressure load (11).

In this study, echocardiography showed that LAD and LAV were significantly enlarged, but LVMI increased slightly after the establishment of the AVF, indicating that the impact of the AVF was not only the adaptive changes caused by increased blood volume but also accompanied by an increase in the LV mass, which is consistent with the results of Ori et al. (12) Volume overload and LV hypertrophy can lead to an increase in LV filling pressure and LV diastolic dysfunction. LA enlargement is a potential marker for most cardiovascular diseases and plays an important role in the risk assessment and prognosis of AF, HCM, and hypertensive heart disease. LAD is also a necessary indicator for evaluating LV diastolic function. Echocardiography is the preferred method for evaluating LAD and LAVI, especially in 2015. According to the American Echocardiography Society's quantitative guidelines, LAVI is the most accurate parameter for evaluating LA size. In many retrospective studies, LAVI information is missing, and only the LAD is included in the echocardiographic reports, a shortcoming shared by our study.

AF is one of the most common arrhythmias, and its mechanism is not yet clear, but it is closely related to congestive heart failure and stroke. Studies have shown that LA enlargement is an independent risk factor for AF (13) and is associated with increased mortality (14). Abnormal LA structure might be an intervention point because LA enlargement often occurs in combination with decreased LA compliance, elevated LA pressure, myocardial interstitial fibrosis, neurohormone metabolism disorder, and subclinical inflammation related to diastolic failure. Our study also found that with increasing LA, the incidence of AF further increased, and age and LA enlargement after AVF establishment were found to be independent risk factors for new-onset AF, which is consistent with the findings of Hassanin N et al. (15) and Reddy et al. (16) Furthermore, as blood dialysis progresses, LA enlargement and dysfunction become more pronounced (17). LA enlargement has been closely related to an increase in thromboembolic events in patients with AF, showing independent predictive value for the occurrence of stroke (18).

AF and heart failure are mutually causative, and atrial excitation during AF cannot be transmitted to the ventricle in a normal rhythm, resulting in abnormal ventricular filling and impaired diastolic function, ultimately leading to AF cardiomyopathy and inducing heart failure (19). When heart failure occurs, acute and chronic LA pressure increase, and increased atrial pressure and enlargement promote scar formation and fibrosis, ultimately leading to conduction abnormalities and inducing AF. A meta-analysis indicated that the risk of heart failure in AF patients increased nearly fivefold (20). Another study showed that mortality due to heart failure in AF patients has increased nearly threefold (21). In this study, the incidence of adverse clinical events (including stroke, heart failure, cardiovascular death, and all-cause mortality) in the AF group was significantly higher than that in the non-AF group, indicating a potentially poor prognosis for AVF haemodialysis patients with concomitant AF. Overall, other factors, such as old age, hypertension, diabetes, hyperlipidaemia, coronary heart disease, obesity, long-term drinking, smoking, family history, lack of exercise and other factors, might play a role in adverse clinical events in MHD patients. CVD is not only the main complication but also the leading cause of death in MHD patients. Studies have shown that persistent AF and heart failure are independent risk factors for all-cause mortality and cardiovascular mortality in nonvalvular AF patients (22). In our study, follow-up revealed that 49 patients (32%) died, with one-third attributed to CVD, which is consistent with previous reports (23, 24). By multifactorial Cox regression correction, new-onset AF after AVF establishment and LV systolic dysfunction were independent risk factors for adverse clinical outcomes in MHD patients, which is consistent with Wang et al. (25)

The treatment of MHD patients with concomitant AF is very complex and controversial, and there are currently no ideal clinical treatment guidelines. In clinical practice, it is often a professional AF management team is recommended to provide personalized treatment for patients. The optimal AF management strategy mainly includes improving patient prognosis (anticoagulation and CVD treatment) and improving symptom treatment (ventricular rate control, rhythm control) (26).

Therefore, regular measurements of cardiac structure and function, including LA size, LV wall thickness, LV systolic and diastolic function, and pulmonary artery pressure, could detect cardiac damage caused by AVF in the early period and draw the attention of doctors and patients as soon as possible, and timely treatment might slow or reverse heart damage. In addition, controlling blood pressure, weight, blood glucose, blood lipids, reducing alcohol consumption, quitting smoking, and reasonable exercise may improve cardiac remodelling and cardiac function to some extent and might reduce the induction of AF development.

## Limitations

This was a single-centre retrospective cohort study with a relatively small sample, and early echocardiographic parameters were inadequate, which prevented further investigation. This study might have some missing clinical data, and paroxysmal AF was difficult to capture, potentially underestimating its real occurrence. Finally, the study overlooked the impact of certain medications, such as ACE inhibitors, anticoagulants, and antiarrhythmic and heart rate-controlling treatments, on CVD. Multicentre studies with more patients and incorporating more parameters will be needed to definitively establish clinical pathways to improve the prognosis of patients on MHD with AVF access.

## Conclusion

LA enlargement and a significant increase in the incidence of AF were observed following AVF establishment in patients with MHD. New-onset AF and LV systolic dysfunction are independent risk factors for adverse clinical outcomes. Active intervention for these risk factors might improve patient prognosis.

## Data Availability

The original contributions presented in the study are included in the article/Supplementary Material, further inquiries can be directed to the corresponding author.
